# Metabolomics reveals signature metabolites associated with high-oiliness flue-cured tobacco (*Nicotiana tabacum L.*) leaves

**DOI:** 10.3389/fpls.2026.1890505

**Published:** 2026-09-04

**Authors:** Xiaodong Wang, Guo Wei, Zhen Gao, Yajie Liu, Shaohua Ge

**Affiliations:** College of Agronomy, Henan University of Science and Technology, Luoyang, China

**Keywords:** biomarkers, carbon-nutrient balance, flue-cured tobacco, oiliness, oxylipin cascade, phenylpropanoid pathway, widely targeted metabolomics

## Abstract

**Background:**

High oiliness is a crucial trait evaluated in flue-cured tobacco (*Nicotiana tabacum L*.) after the curing process, determining its visual appeal and industrial usability. However, its specific metabolic characteristics and objective evaluation markers remain unclear.

**Methods:**

In this study, widely targeted metabolomics and conventional chemical analyses were integrated to systematically profile flue-cured leaves with varying oiliness levels. Results: We identified robust candidate biomarker panels--comprising 20 and 21 characteristic metabolites for middle and upper leaves, respectively--that effectively distinguish the high-oiliness phenotype. Interestingly, our results revealed distinct positional differences in the metabolic signatures associated with high oiliness. In middle leaves, high oiliness was predominantly characterized by elevated soluble sugars and a significant enrichment of phenylpropanoid-derived aroma precursors (e.g., coniferin and syringin), alongside a concomitant accumulation of chloride. Conversely, high-oiliness upper leaves exhibited a distinct signature of lipid remodeling and oxylipin accumulation represented by 9-OxoODE and 12-oxo-PDA; these metabolites were significantly negatively correlated with starch content. Receiver operating characteristic (ROC) analysis validated the high classification accuracy of these candidate biomarkers.

**Conclusion:**

Although further mechanistic validation is required, this study elucidates the complex metabolic profiles of high-oiliness tobacco leaves, providing an objective biochemical evaluation framework and molecular candidate targets for standardized post-curing tobacco grading and precision breeding of high-oiliness varieties.

## 1 Introduction

Flue-cured tobacco (*Nicotiana tabacum L*.) is a high-value economic crop whose economic value is primarily determined by its sensory and smoking quality. In the global tobacco trade and processing industry, “oiliness” is regarded as a critical grading criterion that directly determines the industrial usability of tobacco leaves (Wang et al., 2007; Jing et al., 2022). High-oiliness leaves are phenotypically characterized by a vibrant luster, dense texture, and excellent elasticity, and are internally abundant in aroma precursors and lipid compounds (Hu et al., 2025b; Li et al., 2026). In industrial applications, they typically exhibit superior processing characteristics, including excellent combustibility, optimal filling power, and a harmonious balance between aroma and smoke smoothness (Liu et al., 2020; Hu et al., 2025b). Conversely, low-oiliness (dry) leaves often appear dull and brittle, producing a harsh, thin smoke with a significant lack of aromatic intensity (Hu et al., 2025b; Li et al., 2026). Currently, the evaluation of tobacco oiliness relies heavily on the subjective experience of graders during visual and tactile inspections, and this assessment lacks objective molecular-level diagnostic markers. This has become a major bottleneck hindering the standardized cultivation and precision breeding of high-oil varieties (Meng et al., 2022; Zhang et al., 2026).

Tobacco oiliness is not determined by a single chemical compound but is a coordinated outcome of complex metabolic networks (Zhou et al., 2020). Its formation is closely associated with diverse biochemical components, including epicuticular waxes, endogenous lipids, and glandular trichome exudates (Weimin et al., 2010). These substances serve as both the foundational structural components of the oily phenotype and functional products of plant metabolic regulation; their accumulation levels characterize the allocation efficiency of photosynthetic carbon from primary growth metabolism to specialized quality-related metabolic pathways (Vanhercke et al., 2019; Chu et al., 2021). Furthermore, secondary metabolites such as flavonoids and phenylpropanoids are intimately linked to leaf quality and participate in the synthesis and metabolic regulation of oil-related substances (Li et al., 2025). Additional research indicated that endogenous lipids serve as precursors for the oxylipin cascade--a signaling pathway that can respond to variations in field microenvironments, thereby reshaping the overall metabolic profile of leaf tissue and indirectly influencing the formation and accumulation of tobacco oiliness (Shakir, 2025). Given the chemical complexity of tobacco leaves and the subtle variations between oiliness grades, traditional targeted analysis focusing on only a few major components (e.g., total sugars and alkaloids) is insufficient to decipher the material basis underlying oiliness formation (Adeniji et al., 2023).

Metabolomics, as a high-throughput research tool, has demonstrated remarkable utility in deciphering complex biological traits (Oliver, 2002; Theodoridis et al., 2011). In crop science, this technology has been widely applied to screen stress-response markers in rice, identify flavor precursors in tea, and analyze nutritional shifts in maize (Alseekh and Fernie, 2018; Guipei et al., 2023; Rajkumari et al., 2023; Yang et al., 2025). Metabolomic profiling has also successfully unraveled diverse complex biological mechanisms in tobacco research; for instance, Li et al. (2026) integrated this technique with plant physiology and transcriptomics to elucidate the mechanisms of tobacco root growth and nicotine synthesis under different nitrogen forms, and Zhang et al. (2023) used it to reveal chemical transformation dynamics in cigar tobacco across different regions and fermentation stages. Additionally, metabolomics has been applied to geographical origin identification (Jing et al., 2024), dynamic monitoring of leaf maturity (He et al., 2015), and the characterization of metabolites associated with conventional sensory quality (Lu et al., 2023; Hao et al., 2024). Although metabolomics has been deeply and extensively applied across multiple research directions in tobacco, a systematic biochemical characterization of the “oiliness” trait—particularly regarding the specific metabolic pathways and key biomarkers associated with this complex phenotype—remains to be elucidated.

To gain a more comprehensive understanding of this trait, this study integrated conventional chemical profiling with a widely targeted metabolomics approach to systematically dissect the high-oiliness phenotype in the elite cultivar ‘Yunyan 87’. Specifically, we aimed to: (1) clarify the chemical coordination and sensory quality characteristics of tobacco leaves across varying oiliness levels; (2) screen a set of candidate metabolite biomarkers to establish an objective biochemical basis for oiliness grading; and (3) elucidate the key metabolic pathways that are associated with oiliness formation.

A significant contribution of this research is facilitating the transition of oiliness evaluation from subjective sensory descriptions to a quantifiable, biomarker-based biochemical framework. By comprehensively profiling the metabolic signatures associated with the oily phenotype, this study provided deeper insights into its underlying material basis. Ultimately, these findings offer valuable molecular targets to inform standardized industrial quality control and precision tobacco production.

## 2 Materials and methods

### 2.1 Site description and experimental design

The field experiment was conducted in 2023 in Jiange County, Guangyuan City, Sichuan Province, China (altitude 550–820 m). The region is characterized by a subtropical humid monsoon climate, with an annual mean temperature of 16.0 °C and total annual precipitation of 1077.5 mm. The flue-cured tobacco cultivar ‘Yunyan 87’ was used as the research material. The experimental soil was purple lime soil (pH 6.5) with the following baseline nutrient contents: alkali-hydrolyzable nitrogen 102.00 mg/kg, available phosphorus 13.80 mg/kg, available potassium 268.90 mg/kg, and organic matter 13.20 g/kg.

### 2.2 Sample collection and grading

Following the curing process, the flue-cured tobacco samples were classified in accordance with the enterprise standard of China Tobacco Hubei Industrial Co., Ltd., titled “High oil content tobacco leaves from Medium sized tobacco: Definition, evaluation methods, and quality requirements for raw tobacco”. An evaluation panel consisting of three or more professional quality inspectors quantitatively assessed the physical and tactile characteristics of the leaves using a standardized scoring system. Based on the evaluation scores, the tobacco samples were categorized into three distinct oiliness levels: high (H), medium (M), and low (L).

In this study, middle leaves (C, 10th-12th stalk positions) and upper leaves (B, 16th-18th stalk positions) were selected, and six experimental groups were established: COH, COM, and COL (corresponding to high-, medium-, and low-oil-grade middle leaves, respectively); BOH, BOM, and BOL (corresponding to high-, medium-, and low-oil-grade upper leaves, respectively). Raw tobacco leaves were harvested from different individual plants within multiple field plots. After uniform flue curing treatment, leaves with the same stalk position and identical oil grade level were thoroughly pooled to form one composite sample per group. Subsequently, six 2-kg subsamples were randomly drawn from each pooled composite sample to serve as technical replicates for sample pretreatment and metabolomic detection.

Upon collection, the midribs and large secondary veins were removed, and the laminae were sealed in aluminum foil. Each sample was divided into two subsamples: one was stored at -80 °C for conventional chemical analysis and metabolomic detection, and the other was reserved for further analysis.

### 2.3 Conventional chemical analysis

Tobacco leaf samples (500 g each) of middle and upper stalk positions with different oil levels were collected after curing. The main veins were removed, and the samples were dried to constant weight in a forced-air oven at 50 °C, then ground and sieved through a 0.25 mm mesh. The contents of total sugars, reducing sugars (Administration, 2019), total alkaloids (Administration, 2002a), total nitrogen (Administration, 2002b), potassium (Administration, 2003) (flame photometry), and chlorine (Administration, 2011) were determined using a continuous flow analyzer(Auto Analyzer 3, Seal, Germany). The sugar–alkaloid ratio(total sugars/nicotine), sugar ratio(total sugars/reducing sugars), nitrogen–alkaloid ratio(total nitrogen/total alkaloids), and potassium–chlorine ratio (K^+^/Cl^−^) were calculated accordingly.

### 2.4 Metabolite extraction

Briefly, 50 mg of flue-tobacco leaf powder was weighed into a 2 mL centrifuge tube containing a 6 mm grinding bead. Subsequently, 400 μL of pre-cooled extraction solvent [methanol:water = 4:1 (v/v)], containing 0.02 mg/mL L-2-chlorophenylalanine as an internal standard, was added. The mixture was homogenized using a frozen tissue grinder at -10 °C and 50 Hz for 6 min, followed by ice-bath ultrasonic extraction at 5 °C and 40 kHz for 30 min. The extracts were then incubated at -20 °C for 30 min to facilitate protein precipitation and subsequently centrifuged at 13,000 × g and 4 °C for 15 min. The resulting supernatant was carefully transferred to an autosampler vial for LC-MS/MS analysis. To monitor the stability and reproducibility of the analytical system, quality control (QC) samples were prepared by pooling equal volumes of supernatants from all experimental samples. One QC sample was injected after every six experimental samples throughout the instrumental analysis.

### 2.5 UPLC-MS/MS analytical conditions

Metabolomic analysis was performed using a UPLC-Triple TOF system (AB SCIEX). Technical support for metabolite detection and library matching was provided by the Majorbio Cloud Platform (https://cloud.majorbio.com).

Chromatographic Conditions: Separation was achieved on an HSS T3 column (100 mm × 2.1 mm, 1.8 μm) maintained at 40 °C with a flow rate of 0.4 mL/min. Mobile phase A consisted of 95% water and 5% acetonitrile (containing 0.1% formic acid), and mobile phase B was 47.5% acetonitrile, 47.5% isopropanol, and 5% water (containing 0.1% formic acid). The gradient program was: 0–0.1 min, 0%–5% B; 0.1–2 min, 5%–25% B; 2–9 min, 25%–100% B; 9–13 min, 100% B; 13–13.1 min, 100%–0% B; and 13.1–16 min, 0% B.

Mass Spectrometry Conditions: MS detection used electrospray ionization (ESI) in both positive and negative modes with a scan range of m/z 50–1000. Parameters were: ion spray voltage (+5000 V/-4000 V), declustering potential 80 V, curtain gas 30 psi, ion source gas 1 and 2 both at 50 psi, source temperature 550 °C, and rolling collision energy of 40 ± 20 V.

### 2.6 Data pre-processing and metabolite annotation

Raw LC-MS/MS data were imported into Progenesis QI software (Waters) for peak extraction, alignment, and normalization. The qualitative annotation of metabolites was performed in reference with the Metabolomics Standards Initiative (MSI) guidelines. Specifically, the identification confidence was categorized into two levels: Level B(i), representing the highest identification accuracy, relies on exact MS/MS spectral matching against an in-house library, with a score threshold of ≥ 35; Level B(ii) relies on exact matching against in silico MS/MS spectra from public or theoretical databases, with a score threshold of ≥ 40. Only identification results meeting these criteria were retained. Subsequently, data quality control was performed: metabolite features undetected in > 80% of samples were excluded. Missing values were imputed using the K-nearest neighbor (KNN) algorithm, and variables with a relative standard deviation (RSD) > 30% in QC samples were removed. The retained data were subjected to total ion intensity normalization, Log2 transformation, and Pareto scaling prior to multivariate statistical analysis.

### 2.7 Data analysis

Principal component analysis (PCA) was used to evaluate overall metabolic distribution and reproducibility. Partial least squares discriminant analysis (PLS-DA) was applied to identify metabolic differences among groups. To evaluate the robustness and avoid overfitting of the PLS-DA model, permutation tests (n = 200) (Szymańska et al., 2012) and cross-validation were performed. The model quality was assessed using R²Y and Q² values.

Differentially accumulated metabolites (DAMs) were identified based on VIP ≥ 1, |log2FC| ≥ 1, and FDR < 0.05. The FDR was controlled using the Benjamini–Hochberg method to correct for multiple comparisons.

DAMs were functionally annotated and mapped using the KEGG database (http://www.kegg.jp/kegg/). Metabolic pathway enrichment analysis (MSEA) was performed using a hypergeometric test. P-values were adjusted using the Benjamini-Hochberg method, and pathways with FDR < 0.05 were considered significantly enriched.

Analysis of variance (ANOVA) was performed using SPSS 27.0. Data visualization was conducted using Origin 2024 and Excel 2019.

## 3 Results

### 3.1 Differences in conventional chemical components and their coordination among tobacco leaves with different oiliness levels

**Figure 1:**
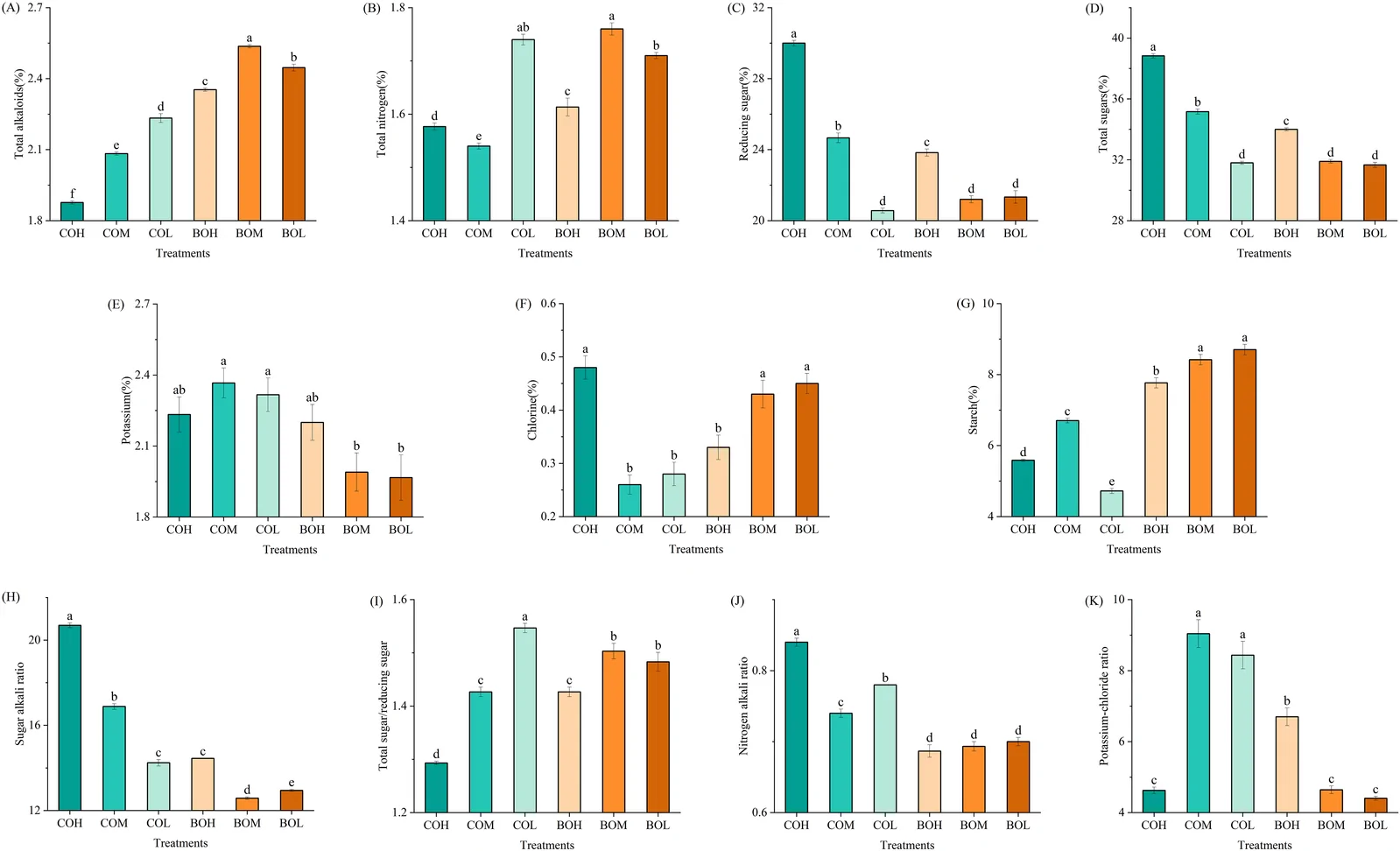
Conventional chemical components and their coordination characteristics in tobacco leaves with different oiliness levels. **(A)** Total alkaloids; **(B)** Total nitrogen; **(C)** Reducing sugar; **(D)** Total sugar; **(E)** Potassium; **(F)** Chloride; **(G)** Starch; **(H)** Sugar-alkaloid ratio; **(I)** Total sugar-reducing sugar ratio; **(J)** Nitrogen-alkaloid ratio; **(K)** Potassium-chloride ratio. Data are presented as mean ± standard deviation. Different lowercase letters indicate significant differences among treatment groups within the same stalk position, based on Duncan’s multiple range test (P < 0.05).

Routine chemical components and derived quality indices serve as the fundamental material basis for evaluating the intrinsic quality and oiliness grading of tobacco leaves. The analytical results (**Figures 1A–G**) demonstrated that chemical components related to carbon-nitrogen metabolism and mineral ions exhibited highly regular and stalk-position-specific patterns across different oiliness grades.

Regarding carbon and nitrogen metabolic indices, a significant gradient variation pattern was observed among the visual oiliness of tobacco leaves and their sugar and alkaloid contents. In both middle and upper leaves, as the visual oiliness grade improved, the contents of total sugars and reducing sugars increased significantly (**Figures 1C, D**), while the total alkaloid content decreased correspondingly (**Figure 1A**). Consequently, high-oiliness tobacco leaves consistently exhibited a distinct “high-sugar and low-alkaloid” chemical phenotype across different stalk positions. In middle leaves, the total sugar (38.83%) and reducing sugar (30.00%) contents of the high-oiliness treatment (COH) were significantly higher than those of the medium-oiliness (COM) and low-oiliness (COL) treatments (P < 0.05). Concurrently, its total alkaloid (1.88%) and total nitrogen (1.58%) contents reached the lowest levels at the same stalk position. Similarly, in upper leaves, the total sugar (34.00%) and reducing sugar (23.83%) contents of the high-oiliness treatment (BOH) were significantly higher than those of BOM and BOL, whereas its total alkaloid (2.35%) and total nitrogen (1.61%) contents remained significantly lower than those of the other treatments at the same stalk position. With respect to starch content, high-oiliness leaves remained at relatively low levels compared to the peak levels observed in other groups at the same leaf position, which ensures good combustibility despite the sharp increase in soluble sugars. The starch content of COH was 5.59%, representing a moderate level within the middle leaves, whereas BOH exhibited the lowest starch content (7.77%), which was significantly lower than that of BOM (8.42%) and BOL (8.70%). Regarding mineral elements, chloride accumulated in a highly stalk-position-specific manner. The chloride content of COH reached the maximum value (0.48%) in middle leaves, being significantly higher than that of COM (0.26%) and COL (0.28%). Conversely, in upper leaves, the chloride content of the high-oiliness treatment (BOH, 0.33%) was significantly lower than that of BOM (0.43%) and BOL (0.45%). Additionally, potassium content varied among treatments, with middle leaves (2.23%-2.37%) being slightly higher overall than upper leaves (1.97%-2.20%).

The chemical coordination indices varied significantly across different oiliness grades (**Figures 1H–K**). As a core indicator for evaluating smoking flavor balance, the sugar-alkaloid ratio of COH was the highest (20.69), which was significantly higher than those of COM (16.88) and COL (14.24); the sugar-alkaloid ratio of BOH (14.45) was also significantly superior to those of BOM (12.58) and BOL (12.95). In middle leaves, COH exhibited the highest nitrogen-alkaloid ratio (0.84), which was significantly higher than COM (0.74) and COL (0.78). Regarding the potassium-to-chloride (K/Cl) ratio, a crucial parameter for tobacco combustibility, COM (9.04) and COL (8.44) in middle leaves were significantly higher than COH (4.63); in upper leaves, BOH exhibited the highest K/Cl ratio (6.70), being significantly higher than BOM (4.64) and BOL (4.40). The two-sugar ratio (total sugar/reducing sugar), reflecting the carbohydrate composition, also showed significant statistical differences across stalk positions and oiliness grades, with COH exhibiting the lowest two-sugar ratio (1.29), presenting a distinct dual specificity driven by both stalk position and oiliness level.

### 3.2 Global metabolic profile analysis of tobacco leaves with different oiliness levels

**Figure 2:**
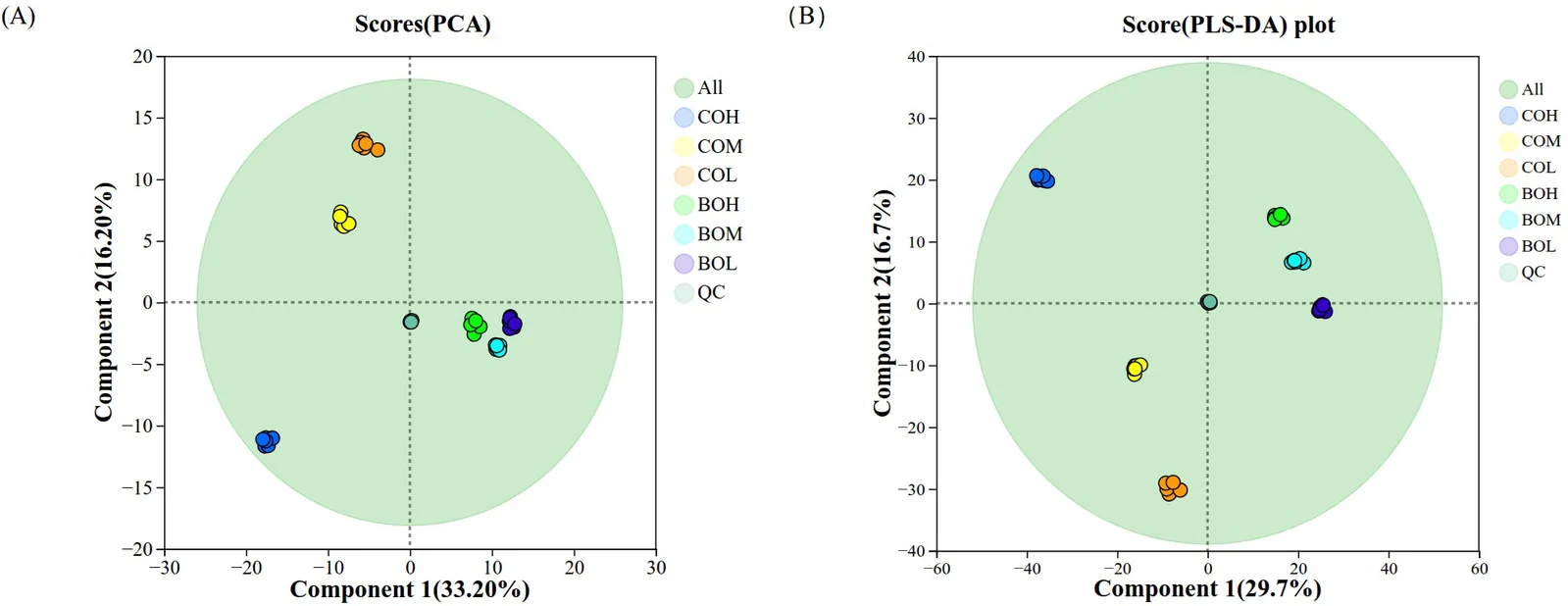
Multivariate statistical analysis of global metabolic profiles in tobacco leaves with different oiliness levels. **(A)** Principal component analysis (PCA) score plot showing overall sample distribution and clustering, including quality control (QC) samples. The first two principal components (Component 1 and Component 2) accounted for 33.20% and 16.20% of the total variance, respectively. **(B)** Partial least squares discriminant analysis (PLS-DA) score plot illustrating the separation of sample groups across different stalk positions and oiliness levels, with Component 1 and Component 2 explained 29.7% and 16.7% of the variance, respectively.

To ensure the reliability of the untargeted metabolomics dataset and the stability of the analytical system, a rigorous quality control assessment was initially conducted on both the quality control (QC) samples and all experimental samples. Initially, a total of 13,574 raw metabolic features were extracted across both positive and negative ion modes. After excluding features undetected in > 80% of samples, an RSD threshold of 30% was applied to the remaining features for quality filtering (Dunn et al., 2011). The Relative Standard Deviation (RSD) distribution plot revealed that 98.81% of the metabolic peaks (retaining 13,194 robust features for downstream analysis) in the QC samples exhibited an RSD of less than 30% throughout the entire LC-MS/MS analytical sequence (**Supplementary Figure 1**). This significantly exceeded the conventional benchmark (typically >80%) (Broadhurst et al., 2018), demonstrating the exceptional reproducibility and stability of the analytical platform during data acquisition. The tight clustering of QC samples in the PCA score space (**Figure 2A**) and the excellent RSD distribution (**Supplementary Figure 1**) comprehensively substantiated this analytical stability.

Concurrently, the sample correlation heatmap (**Supplementary Figure 2**) further supported this conclusion. All QC samples (QC01-QC04) clustered tightly together and displayed extremely high correlation coefficients (r > 0.96), confirming the technical homogeneity of the instrument. Furthermore, biological replicates within each experimental group (e.g., COH1-6, BOH1-6, etc.) exhibited strong positive correlations and distinct intra-group clustering, indicating well-controlled biological variation and high reproducibility of the experimental design.

An unsupervised Principal Component Analysis (PCA, **Figure 2A**) was employed to evaluate the overall data distribution and stability. The results showed that the first two principal components (Component 1 and Component 2) accounted for 33.20% and 16.20% of the total variance, respectively, with a cumulative explanatory rate of 49.40%. The quality control (QC) samples, representing a mixture of all extracts, clustered highly tightly within the PCA score space, fully demonstrating the exceptional stability of the instrument during the analysis and the high reliability of the metabolic data.

To maximize the separation of metabolic differences among different treatment groups, a Partial Least Squares-Discriminant Analysis (PLS-DA) model was further constructed (**Figure 2B**). In this model, Component 1 and Component 2 explained 29.7% and 16.7% of the inter-group variance, respectively. The score plot clearly illustrated that, for both middle and upper leaves, the sample groups of different oiliness levels achieved complete spatial separation within the model space, with tight clustering of intra-group biological replicates. To prevent model overfitting, a 200-fold permutation test was applied to rigorously validate the robustness of the model (**Supplementary Figure 3**). The permutation test results demonstrated that the original model possessed prominent explanatory and predictive capabilities (R^2^X = 0.741, R^2^Y = 0.998, Q^2^ = 0.996). Across the 200 random permutations, the R^2^Y and Q^2^ values of the permuted models were significantly lower than those of the original model, with the permuted Q^2^ intercepts being substantially lower, yielding significance values of P < 0.05 (0/200). This strongly implies that the constructed PLS-DA model is highly robust without any overfitting, lending substantial credibility to the sample classification and the subsequently screened differential metabolites.

Crucially, the score plots revealed distinct clustering driven independently by the two experimental factors: Component 1 (X-axis) primarily captured the impact of stalk position, cleanly separating the middle leaves (clustered on the negative side) from the upper leaves (clustered on the positive side), which indicated that spatial heterogeneity is the dominant driver of metabolic variance. Meanwhile, Component 2 (Y-axis), particularly in the PLS-DA model, successfully resolved the samples along a continuous gradient of oiliness levels from high to low within each stalk position. Together, these quality control and clustering outcomes provided a highly reliable data foundation for the subsequent precise screening of differential metabolites.

The statistical summary of significant differential metabolites (DAMs) across comparison groups is presented in **Table 1**. Based on the global dataset of robust features retained after rigorous quality control, a total of 2,473 metabolites were confidently annotated to specific chemical identities in accordance with the MSI guidelines [Level B(i) and Level B(ii)]. From this high-quality global dataset, a subset ranging from 3764 to 4229 metabolic features was consistently extracted and utilized for each specific pairwise statistical comparison. In middle leaves, the number of DAMs ranged from 680 to 727, with 335 up-regulated and 382 down-regulated features recorded in the COH vs. COM comparison (using COM as the reference baseline). In upper leaves, the number of DAMs for BOH vs. BOM and BOH vs. BOL (similarly using the latter groups as baselines) was 711 and 729, respectively, where the up-regulated features (380 and 401) exceeded the down-regulated ones (331 and 328). The BOM vs. BOL comparison exhibited the highest number of DAMs, totaling 779.

**Table 1:** Statistics of differential metabolites between comparison groups of tobacco leaves with different oiliness grades.

Treatments	No. of valid features	No. of total sig	No. of sig. up	No. of sig. down
COH vs COM	4011	717	335	382
COH vs COL	4229	727	365	362
COM vs COL	4033	680	326	354
BOH vs BOM	3764	711	380	331
BOH vs BOL	4052	729	401	328
BOM vs BOL	4159	779	402	377

Differential metabolites were defined as VIP ≥ 1, |log2FC| ≥ 1, and FDR < 0.05. In all pairwise comparisons (e.g., Group A vs. Group B), expression changes were calculated relative to Group B. ‘Up-regulated’ indicated a significantly higher metabolite abundance in Group A compared to Group B.

### 3.3 KEGG pathway enrichment analysis of differential metabolites across stalk positions

**Figure 3:**
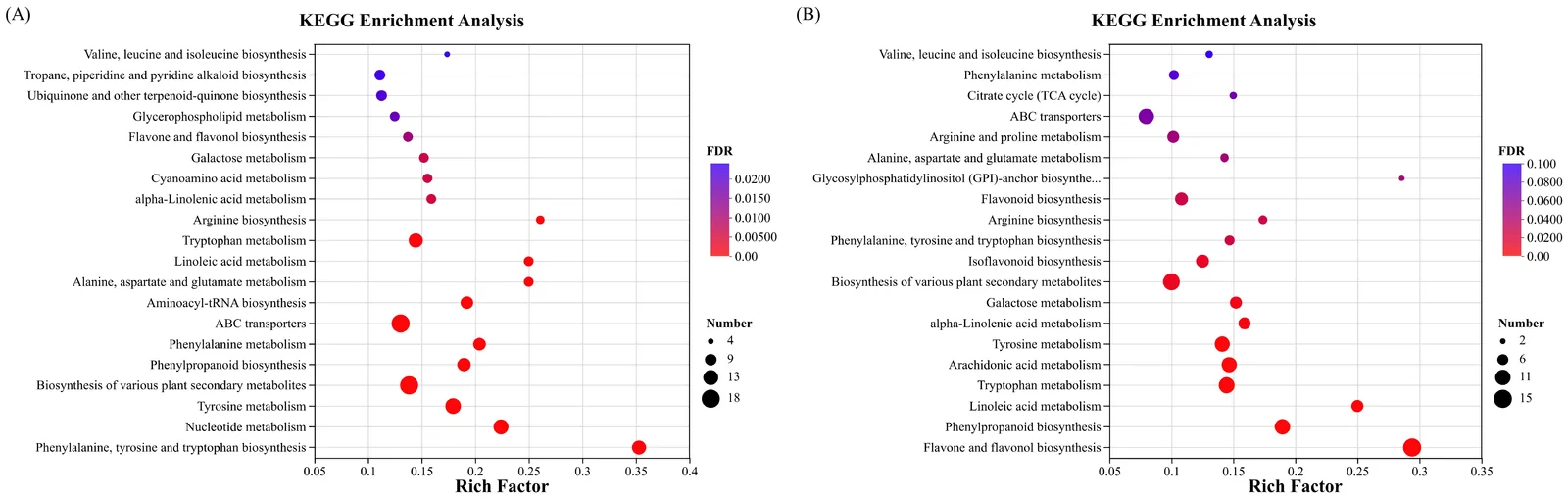
KEGG pathway enrichment analysis of global differential metabolites in tobacco leaves at different stalk positions. **(A)** Middle leaves; **(B)** Upper leaves. Differential metabolites from all comparison groups within the same stalk position were merged and subjected to enrichment analysis. The y-axis represents KEGG pathways, and the x-axis indicates the enrichment ratio (rich factor). Bubble size corresponds to the number of metabolites enriched in each pathway. The color gradient represents the adjusted p-value (FDR) calculated using the Benjamini-Hochberg method.

To comprehensively identify the core pathways associated with tobacco oiliness and enhance the statistical robustness of enrichment analysis, differential metabolites from all comparison groups within the same stalk position were merged for the initial KEGG analysis (**Figure 3**). Subsequently, to elucidate the specific directionality and group-specific biological signals of these metabolites, their relative abundances across different treatments were detailed in the metabolic pathway networks.

The joint enrichment analysis of the differential metabolite set from middle tobacco leaves (**Figure 3A**) demonstrated that the significantly enriched pathways (FDR < 0.05) exhibited strong characteristics of secondary metabolism and aromatic amino acid turnover. The top-ranking pathways were highly concentrated in networks such as Phenylalanine, tyrosine and tryptophan biosynthesis, Phenylpropanoid biosynthesis, Tyrosine metabolism, and Phenylalanine metabolism. Moreover, fundamental pathways including biosynthesis of various plant secondary metabolites, aminoacyl-tRNA biosynthesis, and nucleotide metabolism were significantly enriched. Linoleic acid metabolism was prominently enriched here as well. This global metabolic profile suggested that the differences among various oiliness levels in middle tobacco leaves likely involve the active regulation of the aromatic amino acid pool turnover, which potentially facilitates the subsequent remodeling of complex secondary metabolites (e.g., aroma compounds derived from phenylpropanoids).

Conversely, the joint enrichment results of the differential metabolite set from upper leaves (**Figure 3B**) displayed high spatial heterogeneity, with their core altered pathways being comprehensively “lipid-dominated” (lipidized). In the upper leaves, the highly significantly enriched pathways were almost exclusively occupied by lipid biosynthesis and oxidative degradation routes. These included the core Linoleic acid metabolism, alpha-Linolenic acid metabolism, Arachidonic acid metabolism, and Galactose metabolism, which provided primary carbon source support. Concurrently, pathways such as Tryptophan metabolism, Isoflavonoid biosynthesis, and Arginine biosynthesis successfully ranked among the top significantly enriched lists. The obvious convergence and clustering within the fatty acid metabolic cascades indicate that the formation of the high-oiliness phenotype in upper tobacco leaves was closely associated with the dynamic remodeling of cellular lipid oxidation, seemingly reflecting a coordinated biochemical adjustment process.

### 3.4 Screening of key differential metabolites in high-oiliness tobacco leaves and correlation analysis with conventional chemical components

**Figure 4:**
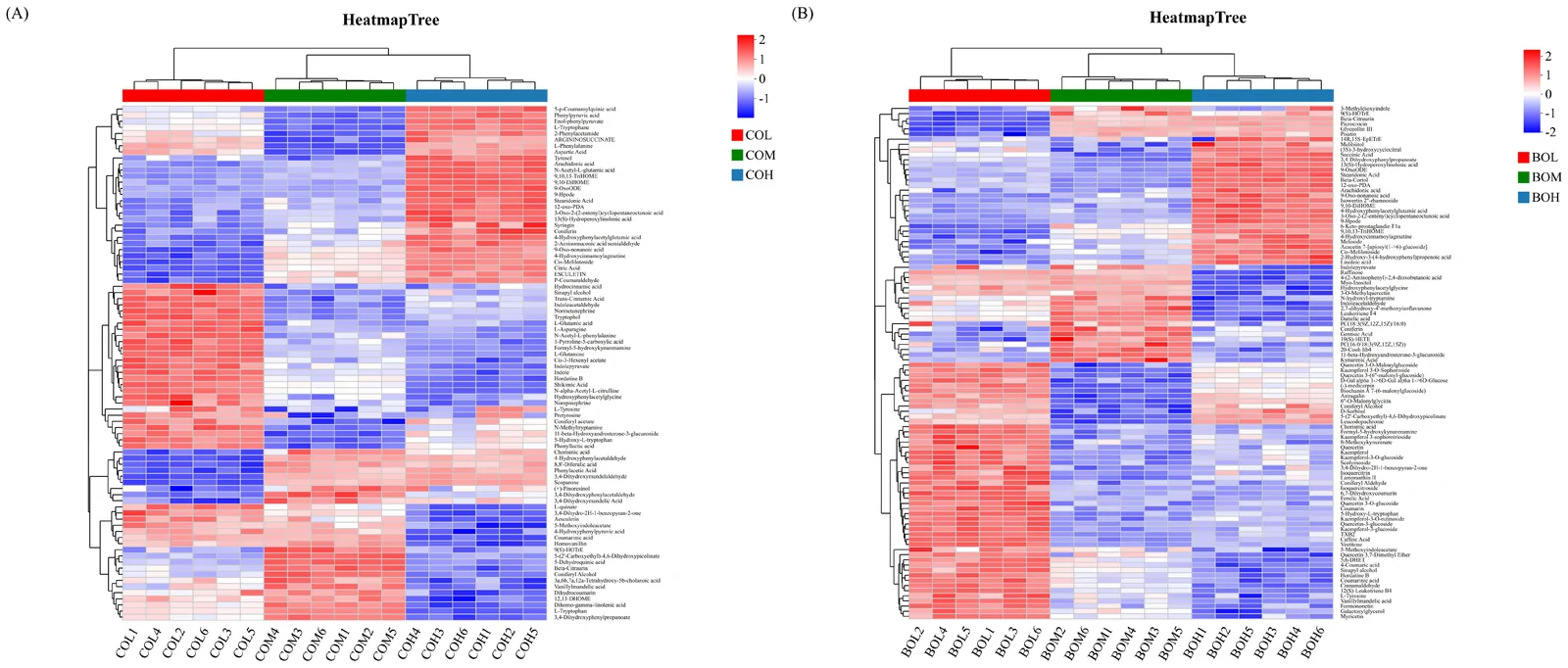
Hierarchical clustering heatmaps of differential metabolites from the top 10 enriched KEGG pathways in tobacco leaves with different oiliness levels. **(A)** Middle leaves; **(B)** Upper leaves. The color scale represents relative metabolite abundance after Z-score normalization, with red indicating higher abundance and blue indicating lower abundance.

To precisely pinpoint the core substances characterizing the high-oiliness trait, this study further extracted all differential metabolites from the top 10 significantly enriched pathways of middle and upper leaves according to the pathway enrichment results in Section 3.3, and performed systematic hierarchical clustering analysis (**Figure 4**).

In the middle leaves (**Figure 4A**), a distinct cluster comprising 20 differential metabolites emerged clearly, exhibiting significant accumulation in the COH group. Based on their biochemical structures, these 20 potential candidate biomarkers could be systematically classified into four major categories: amino acid derivatives and phenylpropanoids (coniferin, syringin, enol-phenylpyruvate, phenylpyruvic acid, L-tryptophane, tyrosol, 5-p-coumaroylquinic acid, and 4-hydroxyphenylacetylglutamic acid); sugars and carbohydrates (myo-inositol, trehalose, and raffinose); lipids and oxylipin signaling molecules (arachidonic acid, 9-OxoODE, 9-Hpode, and 9,10,13-TriHOME); and nucleotides and their derivatives (guanine, guanosine, uridine, and cytosine deoxyribonucleoside).

In the clustering results for the upper leaves (**Figure 4B**), a highly expressed cluster consisting of 21 metabolites was similarly targeted, exhibiting specific significant accumulation in the BOH group. In stark contrast to the middle leaves, these 21 substances were heavily focused on the lipid network and could be divided into three main categories: free fatty acids and oxylipin cascade products (linoleic acid, arachidonic acid, stearidonic acid, 9-oxo-nonanoic acid, oxidized/signaling lipids 9-OxoODE, 9-Hpode, 13(S)-hydroperoxylinolenic acid, 14R,15S-EpETrE, 6-keto-prostaglandin F1a, and the jasmonic acid-like precursor 3-oxo-2-(2-pentenyl)cyclopentaneoctanoic acid); phenolics, flavonoids, and glycoside derivatives (acacetin 7-[apiosyl(1->6)-glucoside], cis-melilotoside, meloside, 2-hydroxy-3-(4-hydroxyphenyl) propenoic acid, 4-hydroxyphenylacetylglutamic acid, and 3,4-dihydroxyphenylpropanoate); and other primary metabolites and terpenoids (including succinic acid, melibiitol, as well as the terpenoid derivative (3S)-3-hydroxycyclocitral and steroids).

Based on the aforementioned classification and expression specificity, these 20 and 21 substances were ultimately confirmed as the candidate biomarker sets characterizing the quality specificity of high-oiliness tobacco leaves in the middle and upper stalk positions, respectively.

To further evaluate the diagnostic efficacy and classification accuracy of the identified differential metabolites in distinguishing the high-oiliness phenotype, a Receiver Operating Characteristic (ROC) curve analysis was performed on the candidate biomarkers from both middle and upper tobacco leaves (the detailed curves are presented in **Supplementary Figure 3**). The results demonstrated that the selected candidate differential metabolites possessed extraordinary classification capacities. For the middle leaves, key characteristic volatile precursors and metabolites, including coniferin, syringin, and L-tryptophane, exhibited excellent diagnostic performance, each achieving an area under the curve (AUC) value of 1.0000 with a 95% confidence interval (CI) of 1.0000-1.0000 (**Supplementary Figure 4A**). Similarly, in the upper leaves, critical lipid-remodeling and stress-signaling biomarkers, such as 9-OxoODE and 12-oxo-PDA, displayed pristine classification potential, yielding AUC values of 1.0000 (95% CI: 1.0000–1.0000). Notably, 14R,15S-EpETrE, which possessed extraordinary classification efficacy, also achieved a maximum AUC value of 1.0000 (**Supplementary Figure 4B**); its numerical confidence interval boundary (0.0000-1.0000) stems from the statistical resampling effect caused by the complete separation (non-overlapping data) of this metabolite between the two study groups.

**Figure 5:**
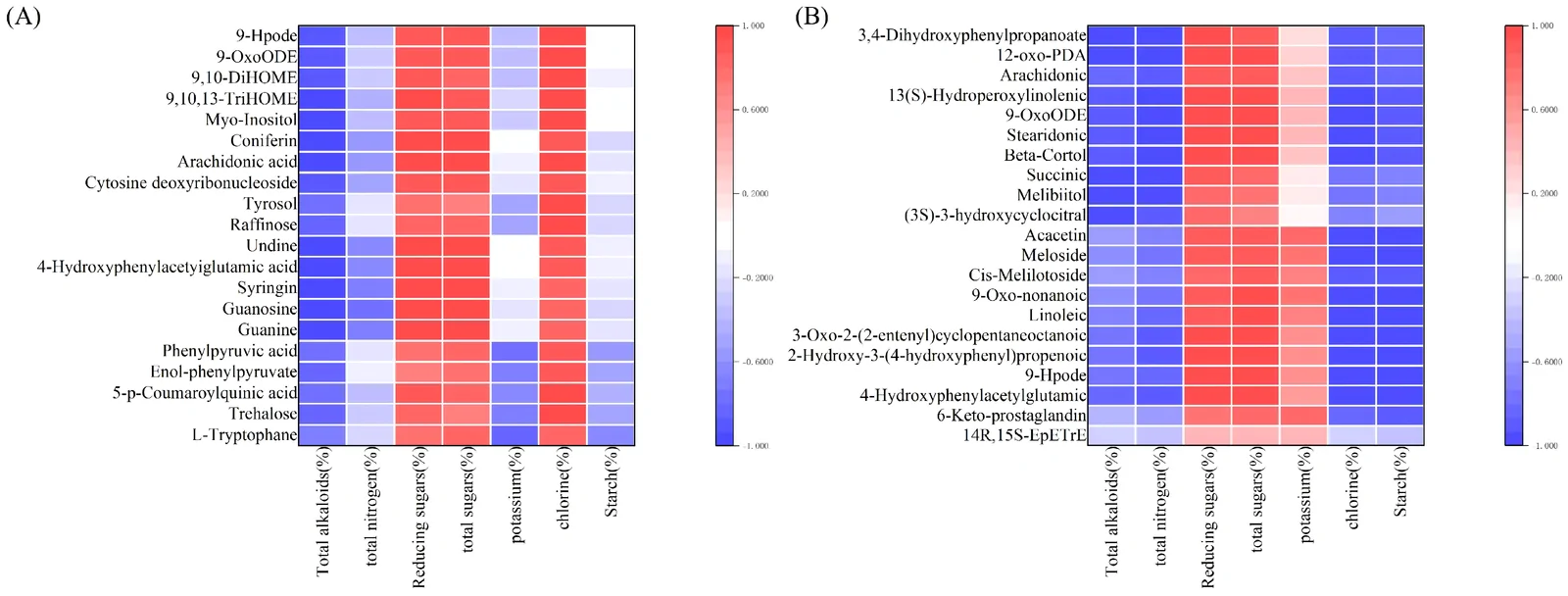
Pearson correlation analysis between key biomarker metabolites and conventional chemical components in tobacco leaves with different oiliness levels. **(A)** Middle leaves; **(B)** Upper leaves. The color scale represents the Pearson correlation coefficient (r), with red indicating positive correlation and blue indicating negative correlation. Statistical significance was assessed based on P-values, with P < 0.05 and P < 0.01 considered significant and highly significant, respectively.

To explore the intrinsic connections between microscopic biomarker metabolites and macroscopic conventional chemical traits in high-oiliness tobacco leaves, a Pearson correlation analysis was conducted between the key differential metabolites of high-oiliness middle and upper leaves and conventional chemical components (**Figure 5**). The correlation results strongly corroborated the conventional chemical features observed in Section 3.1. As shown in **Figure 5A**, the 20 biomarker metabolites identified in middle leaves exhibited highly significant positive correlations (P < 0.01) with total sugar, reducing sugar, and chloride ions, while displaying highly significant negative correlations (P < 0.01) with total alkaloids. Conversely, in the upper leaves (**Figure 5B**), the 21 identified biomarker metabolites also showed significant positive correlations with reducing sugar and total sugar. However, exhibiting position-specificity distinct from the middle leaves, they demonstrated significant negative correlations with total nitrogen, chloride, and starch. Integrating the results from conventional chemical analysis and the metabolite correlation network, this study ultimately suggested that these 20 (middle) and 21 (upper) differential metabolites could bridge the molecular metabolic and macroscopic phenotypic scales, serving as core metabolic biomarkers to characterize the high-oiliness trait of their respective stalk positions.

### 3.5 Pathway enrichment analysis and construction of metabolic networks for high-oiliness tobacco leaves

To systematically elucidate the biochemical basis underlying the quality traits of high-oiliness tobacco leaves, pathway enrichment analysis was performed using the KEGG database based on the identified candidate differential metabolites from the middle (20 metabolites) and upper leaves (21 metabolites), followed by the construction of refined metabolic regulatory networks.

**Figure 6:**
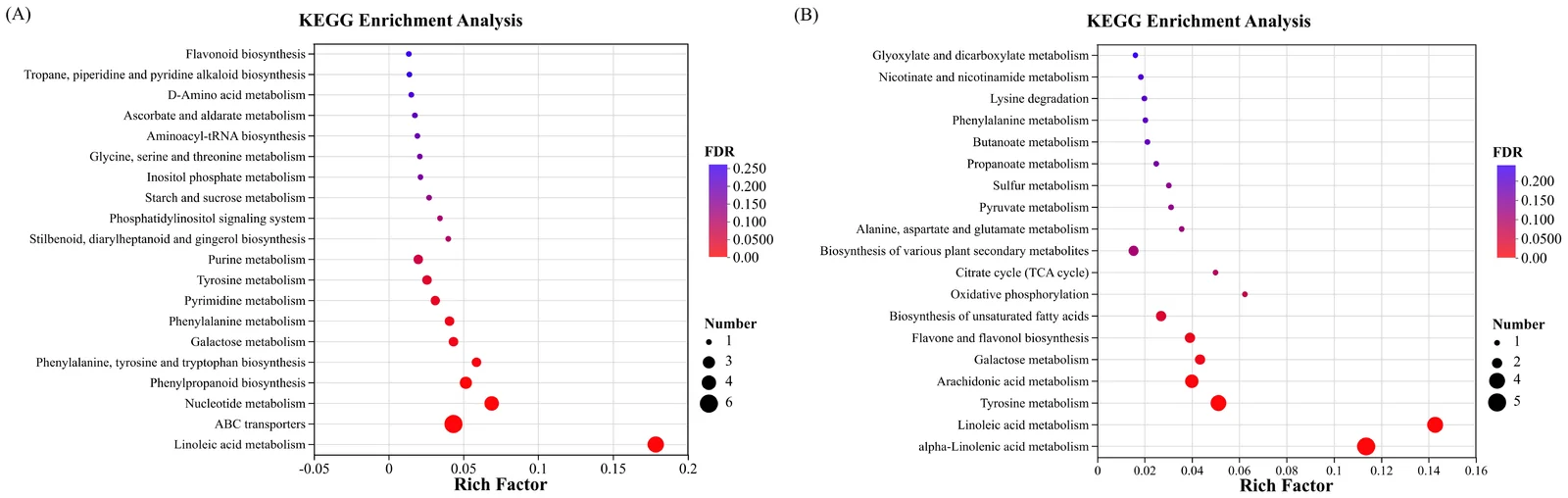
KEGG pathway enrichment analysis of key differential metabolites in high-oiliness tobacco leaves. **(A)** Middle leaves; **(B)** Upper leaves. Bubble size represents the number of metabolites involved in each pathway. The color gradient indicated the adjusted p-value (FDR), with lower FDR values representing higher statistical significance.

**Figure 7:**
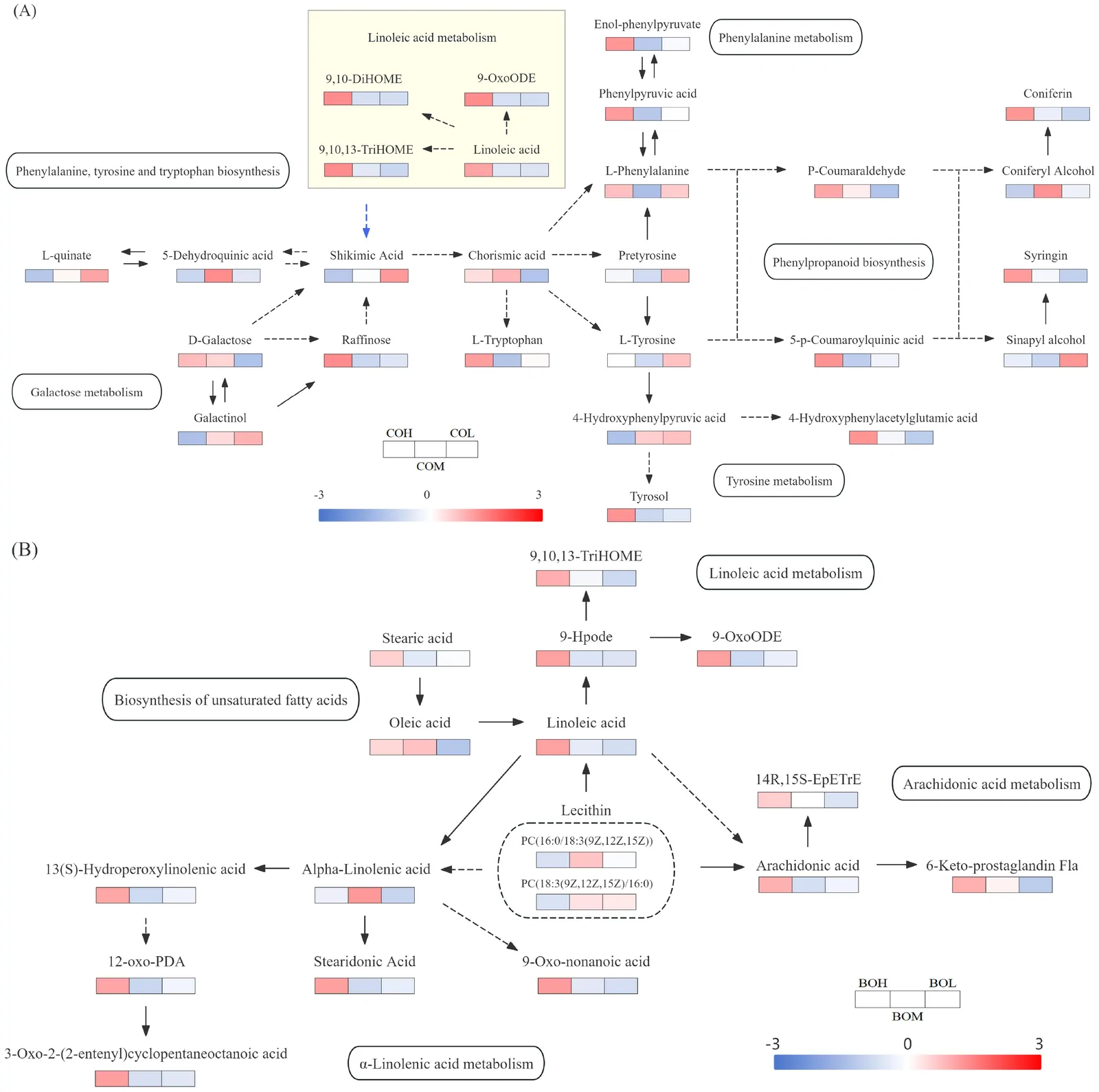
Metabolic pathway networks of key differential metabolites in high-oiliness tobacco leaves. **(A)** Middle leaves; **(B)** Upper leaves. The pathways illustrate the integration of significantly enriched metabolic routes. Bar charts represent relative metabolite abundance across different treatment groups (COH, COM, COL and BOH, BOM, BOL).

The enrichment analysis of differential metabolites in the middle high-oiliness tobacco leaves (COH) (**Figure 6A**) revealed that the significantly enriched pathways (FDR < 0.05) primarily involved linoleic acid metabolism (5 differential metabolites), ABC transporters (6 metabolites), nucleotide metabolism (4 metabolites), phenylpropanoid biosynthesis (3 metabolites), and various amino acid metabolic pathways. During the construction of the core metabolic network (**Figure 7A**), these pathways were critically filtered and integrated. Although the ABC transporters pathway showed the most significant enrichment, it was excluded from the biochemical reaction network as it primarily mediated the transmembrane transport of substrates rather than active biochemical transformations. Similarly, nucleotide and pyrimidine metabolism were omitted because they generally represent basic housekeeping cellular activities and lack the biological specificity required to characterize tobacco leaf quality. Ultimately, a “primary carbohydrate metabolism-aromatic amino acids-phenylpropanoids” metabolic pathway was constructed for the middle leaves. The results showed that, driven by an active primary metabolic background (e.g., galactose metabolism), middle leaves directed metabolic fluxes through the shikimate pathway--a crucial metabolic hub--to directionally drive the turnover of aromatic amino acids such as phenylalanine. At the terminus of this network, precursors of lignin monomers, such as coniferin and syringin, exhibited significant accumulation, indicating that the COH phenotype was closely associated with the active accumulation of secondary metabolites. Additionally, linoleic acid metabolism was incorporated into the pathway, acting as a logical bridge for feedback regulation via lipid signaling.

The pathway enrichment analysis for the upper high-oiliness tobacco leaves (BOH) (**Figure 6B**) presented a metabolic profile distinctly different from that of the middle leaves. The significantly enriched pathways were highly concentrated within the lipid metabolism domain, including α-linolenic acid metabolism (5 compounds), linoleic acid metabolism (4 compounds), arachidonic acid metabolism (3 compounds), and the biosynthesis of unsaturated fatty acids (2 compounds). To delineate the highly specific and biologically relevant metabolic pathways characterizing BOH (**Figure 7B**), the flavone and flavonol biosynthesis pathway was excluded from the network construction, as it contained only two differential products (isovitexin-2’’-O-glucoside and a luteolin glycoside derivative), which were deemed insufficient to represent a global metabolic remodeling. Similarly, non-specific pathways such as galactose metabolism (reflecting primary carbon source responses) and tyrosine metabolism (lacking direct biochemical connections to the core lipid cascades) were also filtered out. Ultimately, an “unsaturated fatty acids–phospholipid pool–oxylipins cascade” metabolic pathway was constructed for the upper leaves. This mapped network originated from the biosynthesis of unsaturated fatty acids (stearic acid → oleic acid → linoleic acid) and converges into a structural and storage lipid pool centered around lecithin. The network mapping indicated that the BOH phenotype is likely associated with the active release of free linoleic and α-linolenic acids from the phospholipid pool, which subsequently facilitates downstream oxylipin metabolism. This dynamic metabolic routing was reflecting by the significant enrichment of corresponding signaling molecules, such as 9-OxoODE, 9,10,13-TriHOME, and the jasmonic acid (JA) precursor 12-oxo-PDA.

## 4 Discussion

### 4.1 Coordination of conventional chemical components and their physiological metabolic mechanisms

When evaluating tobacco quality, the internal coordination of chemical components exerts a more critical and decisive influence than any single isolated indicator (Tian et al., 2025; Wu et al., 2025). According to established chemical evaluation standards for high-quality flue-cured tobacco (Pinfield, 2001; Shi and Liu, 2011), the ideal chemical coordination parameters are generally defined as: a sugar-alkaloid ratio of 10.0-15.0, a reducing sugar-to-total sugar ratio of ≥0.80, a nitrogen-alkaloid ratio of 0.5-1.0, and a potassium-chlorine ratio of ≥4.0. Based on our findings, high-oiliness leaves demonstrated exceptional potential for chemical coordination. In this study, high-oiliness middle tobacco leaves (COH) exhibited the highest chloride ion content (0.48%) and the lowest potassium-to-chloride (K/Cl) ratio (4.63) among the same leaf positions. From a plant physiological perspective, tobacco is a characteristic Cl-rich plant, and moderate chloride accumulation represents an energetically efficient osmoregulation strategy (Franco-Navarro et al., 2016; Colmenero-Flores et al., 2019). The relatively abundant accumulation of chloride ions in COH physiologically balances the intracellular osmotic pressure fluctuations induced by the extremely high accumulation of soluble sugars (total sugar content of 38.83%) (Franco-Navarro et al., 2021; Wu et al., 2024). Through vacuolar compartmentalization, this coordinated deployment of inorganic anions not only sustains cell water potential and turgor under a “high-sugar” microenvironment but also conserves carbon and nitrogen skeletons for secondary metabolite biosynthesis (Rosales et al., 2020; Herrera et al., 2025).

Furthermore, tobacco combustibility does not depend solely on the absolute concentration of chloride ions but is tightly governed by the K/Cl ratio. Theoretically, potassium ions catalyze the complete combustion of carbon, whereas chloride ions retard combustion due to their high electronegativity (Myhre et al., 1956; Bozhinova et al., 2026). Studies have confirmed that a K/Cl ratio greater than 4 guarantees desirable burning capacity (Chen et al., 2025; Yao et al., 2025). In our trial, the COH group concurrently accumulated higher levels of potassium ions (2.23%) alongside chloride, successfully maintaining the K/Cl ratio at 4.63, which is above the critical safety threshold. This dynamic equilibrium of potassium and chloride ions effectively counteracts the potential negative impact of chloride on combustibility, while establishing a stable intracellular ionic homeostasis required for the subsequent biosynthesis of key oiliness precursor substances, such as phenylpropanoids (Colmenero-Flores et al., 2019; Wu et al., 2024).

On the other hand, the sugar ratio (total sugar/reducing sugar) serves as a core coordination indicator reflecting carbohydrate composition and conversion efficiency (Faculty of Food Technology Osijek, 2020). Our data indicated that while the COH group possessed the highest overall sugar content, its sugar ratio dropped to the lowest among all groups (1.29). Mechanistically, a lower sugar ratio demonstrates that non-reducing sugars (such as sucrose) underwent more thorough enzymatic hydrolysis during leaf maturation and curing, leading to massive conversion into reducing sugars (Meng et al., 2022). Furthermore, this disproportionate accumulation may also be driven by the physiological shifts during the curing process, where active cellular respiration and early Maillard reactions preferentially consume reducing sugars (Banožić et al., 2020), while dehydration stress could induce the relative accumulation of non-reducing sugars, such as sucrose, acting as osmoprotectants (Sami et al., 2016). Reducing sugars not only directly impart a sweet and mellow sensation to the smoke but also act as crucial precursors for high-quality aroma compounds. Under the high temperatures of smoking, abundant free reducing sugars react more efficiently with free amino acids via the Maillard reaction, generating heterocyclic volatile aromatic compounds such as pyrazines and furans (Hu et al., 2025a), thereby endowing the high-oiliness middle leaves with an excellent smoking flavor profile.

It is worth noting that the statistical differences observed in the conventional chemical composition of high-oiliness leaves are not merely numerical variations, but hold deep biological and industrial significance. Biologically, these chemical profiles directly reflect the efficiency of enzymatic reactions (such as starch hydrolysis) and the balance of cellular respiration during the curing and senescence processes (Meng et al., 2022; Zhang et al., 2023). From an industrial perspective, the coordinated balance of these components serves as a vital criterion for assessing tobacco quality (Weimin et al., 2010). Specifically, optimal sugar-to-alkaloid and potassium-to-chloride ratios dictate smoke mildness and combustibility (Myhre et al., 1956). Furthermore, these coordinated chemical signatures are crucial for the Maillard reaction and the formation of the distinctive aroma style associated with high-oiliness tobacco (Hu et al., 2025a, 2025b), ultimately providing data-driven guidance for industrial leaf blending.

### 4.2 Carbon allocation mechanisms and metabolic logic of high-oiliness tobacco leaves based on the CNB hypothesis

The macroscopic chemical composition of tobacco leaves is a direct manifestation of the internal metabolic homeostasis of the plant (Faculty of Food Technology Osijek, 2020). In this study, high-oiliness tobacco leaves (COH and BOH) predominantly manifested a chemical profile characterized by “high sugar and low alkaloids.” According to the Carbon-Nutrient Balance (CNB) hypothesis, when nitrogen assimilation is insufficient to support rapid primary growth while photosynthetic carbon fixation remains relatively surplus, the excess carbon flux is preferentially channeled into the biosynthesis of various carbon-rich secondary metabolites (Bryant et al., 1983; Fritz et al., 2006; Zhou et al., 2020; Liu et al., 2025). Our experimental dataset aligns strongly with this hypothesis: the soluble sugar content in high-oiliness leaves was significantly elevated without a concurrent accumulation of starch, indicating that these soluble sugars likely flowed primarily into active metabolic turnover and secondary metabolic partitioning rather than being converted into starch for static storage (Chu et al., 2021). Concurrently, the lower alkaloid content to some extent reflects a shift in the internal carbon-to-nitrogen allocation ratio, which may favor the diversion of more carbon molecules away from primary growth metabolism and toward the synthesis of carbon-rich compounds, such as phenylpropanoids and lipids (Fritz et al., 2006; Meng et al., 2022). This directional adjustment in resource allocation may provide the material foundation for the formation of leaf oiliness and exert a positive influence on the subsequent smoking flavor characteristics of the tobacco leaves (Li et al., 2026).

### 4.3 High-oiliness middle leaves: sugar-signaling-induced enrichment of phenylpropanoid aroma precursors

In this study, middle high-oiliness tobacco leaves (COH) exhibited a global enrichment trend for metabolites associated with the phenylpropanoid pathway. The elevated levels of soluble sugars in COH samples could provide abundant carbon substrates for phenylalanine biosynthesis via the shikimate pathway. Studies have confirmed that the accumulation of intracellular soluble sugars are highly associated with the promotion of phenylalanine ammonia-lyase (PAL)-related metabolic pathways, thereby facilitating an increased synthetic flux of phenylpropanoids (Fritz et al., 2006; Le Roy et al., 2016; Moran et al., 2021). In our experiment, the contents of two candidate biomarkers screened from COH--coniferin and syringin--were significantly increased. This trend demonstrated that when an internal leaf carbon-to-nitrogen imbalance occurs, the excess carbon flux is more inclined to be converted into phenylpropanoid derivatives (Lu et al., 2023). Although coniferin and syringin themselves are non-volatile, they are considered indispensable aroma precursors during the smoking process (Ito et al., 2000). Under high-temperature pyrolysis conditions, these two glycosidic substances could be converted into neutral aroma components such as eugenol, guaiacol, and vanillin. These phenolic compounds helped mitigate the localized harshness of smoke and reduce throat irritation, which may correlate intrinsically with the mellow and sweet smoking sensory experience typical of middle leaves (Baker and Bishop, 2004; Yuan et al., 2014; Jiang et al., 2025).

### 4.4 High-oiliness upper leaves: stress-induced lipid remodeling and the “oxylipin cascade”

Unlike middle leaves, upper tobacco leaves develop at the top of the plant canopy, frequently encountering combined environmental factors such as intense light radiation, high transpiration, and moisture fluctuations. The reduction in starch content within BOH samples suggested that the emphasis of leaf carbon metabolism may have shifted: less carbon was locked into polysaccharide (starch) storage, whereas more carbon sources were mobilized toward the synthesis of lipid secondary metabolites, which might be related to maintaining cell membrane stability and adapting to the external environment. This adjustment in metabolic partitioning improves the visual oiliness features while also helped to avoid the burnt bitterness and off-flavors caused by excessive starch combustion (Chu et al., 2022; Shakir, 2025; Li et al., 2026; Zhang et al., 2026). Furthermore, the substantial enrichment of characteristic lipid metabolites such as 9-OxoODE, 9-Hpode, and 12-oxo-PDA in BOH highlighted that the lipoxygenase (LOX)-mediated metabolic cascade is highly activated in high-oiliness upper leaves. As a direct intermediate of unsaturated fatty acid oxidation, the upregulation of 9-Hpode to some extent reflects active internal lipid remodeling reactions within the leaves (Feussner and Wasternack, 2002). Meanwhile, the accumulation of 12-oxo-PDA, an important precursor in the jasmonic acid (JA) signaling pathway, implied that upper leaves might initiate corresponding hormonal regulations in response to their habitat (Wasternack and Hause, 2013). These lipid metabolic processes might alter the cell membrane composition of leaves while simultaneously increasing the diversity of tissue-specific lipid derivatives (Weber, 2002). These variations in lipid composition might influence the smoking quality of BOH via multiple mechanisms. On a physical level, the enriched long-chain fatty acids and their derivatives could provide a degree of lubrication and buffering during combustion, physically mitigating the harshness and throat-scratching pungency typical of upper canopy leaves to render the smoke smoother and fuller (Li et al., 2026). On a chemical level, these diverse lipid precursors generated via the LOX pathway undergo oxidative pyrolysis upon heating, yielding a series of mild volatile aldehydes and ketones, such as nonanal and decanal, which enrich the overall aroma profile (Wang et al., 2007; Moldoveanu, 2010).

### 4.5 Synthesis of spatial metabolic heterogeneity and perspectives on future absolute quantification validation

In summary, the phenotypic traits of high-oiliness tobacco leaves are not determined by a uniform metabolic blueprint but rather exhibited strong spatial heterogeneity across different leaf positions. High oiliness in middle leaves is predominantly characterized by the accumulation of phenylpropanoid aroma precursors derived from surplus carbon flux, whereas the metabolic foundation of upper leaves is tightly linked to stress-responsive lipid remodeling and the lipoxygenase cascade. Notably, the distinct signature metabolomes identified in the middle and upper leaves are collectively shaped by two layers of factors: intrinsic physiological traits formed during field cultivation, and the coordinated biochemical transformations triggered by standardized flue-curing procedures. Since all leaf samples in this study underwent identical curing protocols, the differential metabolite panels we identified accurately reflect the final oiliness phenotype evaluated by industrial grading standards. Thus, they can serve as objective biochemical markers representing authentic post-curing tobacco quality for industrial applications.

Importantly, it must be noted that the current study has two objective limitations. First, the candidate biomarker data (e.g., coniferin, syringin, 9-Hpode, and 12-oxo-PDA) were captured via untargeted metabolomics, meaning that the dataset reflects relative abundance rather than absolute concentrations, which limits the determination of exact physiological thresholds. Additionally, while some candidate biomarker metabolites exhibited mathematically perfect classification (e.g., AUC = 1.000) in the current dataset, these findings were not validated using an independent external cohort. Crucially, the plant metabolome is highly sensitive to environmental fluctuations. The tobacco leaf samples utilized in this study were sourced from a single specific geographical region and a single harvest year. Consequently, the distinct metabolic signatures and the extraordinarily high classification performance of the identified candidate biomarkers may exhibit significant dataset dependency, largely driven by the localized ecological conditions (e.g., specific climate, soil physicochemical properties, and rainfall) of that particular year. Therefore, the excellent discriminatory power of these metabolites is currently confined to the present specific conditions, and they should be regarded as putative biomarkers. Future large-scale trials covering different seasons, multiple years, and diverse ecological regions should be performed to dissect genotype-by-environment (G×E) interactions, combined with targeted absolute quantification analysis. These efforts would help improve the applicability of these candidate biomarkers. Furthermore, the metabolic flux variations and lipid remodeling mechanisms proposed in this study are primarily deduced from correlative analyses of metabolite abundances. Since this study does not provide corresponding enzyme activity assays (e.g., PAL, LOX) or gene expression data (e.g., qPCR or transcriptomics), and lacks targeted quantification of stress-related hormones (such as ABA and JA), there are inherent limitations in establishing definitive causal relationships for these molecular regulatory pathways. Second, the metabolic regulation models proposed in this study--namely, the “primary carbohydrate metabolism-aromatic amino acids-phenylpropanoids” axis and the “unsaturated fatty acids-phospholipid pool-oxylipins” cascade--are primarily deduced from static metabolite distribution patterns and correlation networks. Lacking direct functional validation through dynamic approaches such as isotope tracing or transcriptomics, the kinetic flux of these pathways remain largely inferential at this stage. Therefore, in subsequent validation phases, it is imperative not only to introduce authentic chemical standards for rigorous absolute quantification using targeted metabolomics, but also to incorporate metabolic flux analysis and multi-omics integration in future studies. Such approaches would dynamically and genetically validate the activity of these signature pathways, thereby providing a more solid data foundation for the molecular breeding and precise agronomic regulation of high-oiliness traits in tobacco.

## 5 Conclusion

In this study, we integrated conventional chemical analysis with widely targeted metabolomics to objectively evaluate the “oiliness” trait in flue-cured tobacco leaves. Our results indicated that high-oiliness leaves generally exhibit a “high-sugar, low-alkaloid” macrochemical phenotype. Furthermore, we identified clear position-specific metabolic signatures associated with this trait, screening 20 and 21 candidate biomarker metabolites for middle and upper leaves, respectively. Metabolite abundance patterns suggested that oiliness in middle leaves was strongly associated with the accumulation of end-products in the shikimate and phenylpropanoid pathways. In contrast, oiliness in upper leaves was linked to lipid remodeling and changes in the abundance of metabolites within the oxylipin cascade. However, several limitations to this study must be acknowledged. First, the mechanistic associations proposed herein are based on metabolite abundance correlations and currently lack validation through enzymatic activity assays (e.g., PAL, LOX) or gene expression data. Second, the widely targeted metabolomics approach primarily provided putative identifications; therefore, absolute quantification using authentic standards was required for future validation. Despite these limitations, our findings offer novel insights into the metabolic basis of tobacco leaf oiliness and provide valuable position-specific targets for future marker-assisted breeding and agricultural quality control.

## Data Availability

The original contributions presented in the study are included in the article/**Supplementary Material**. Further inquiries can be directed to the corresponding author.
