# Supplementary material for: Metabolomics reveals signature metabolites associated with high-oiliness flue-cured tobacco (Nicotiana tabacum L.) leaves
Source: Front Plant Sci. 2026 Sep 4;17:1890505. doi: 10.3389/fpls.2026.1890505 (PMC13585713; doi:10.3389/fpls.2026.1890505)
Supplement: Supplementary file 1 [file DataSheet1.zip › File S1.pdf]

Enterprise Standard of Hubei Tobacco Industry Co., Ltd

QB/HBZY. CL25.1-2020

High oil content tobacco leaves from Medium sized tobacco: Definition, evaluation methods, and quality requirements for raw tobacco

Released on December 30, 2020 and implemented on February 1, 2021

Hubei Tobacco Industry Co., Ltd. releases

preface

Based on the high-quality development of the "Yellow Crane Tower" brand and core raw material requirements, the development of high oil tobacco leaves from Medium sized Tobacco has been implemented, and the " Medium sized Tobacco High Oil Tobacco Leaves" series of standards have been specially formulated.

The book ' Medium sized Tobacco High Oil Tobacco Leaves' consists of 8 parts:

——Part 1: Definition, Evaluation Methods, and Original Tobacco Quality Requirements;

——Part 2: Varieties and Ecological Regions;

——Part 3: Field growth and appearance;

——Part 4: Key Cultivation Techniques;

——Part 5: Baking and Storage;

——Part 6: Grading Technical Requirements;

——Part 7: Leaping and re baking;

——Part 8: Aging maintenance.

This section is the first part of "High Oil Tobacco Leaves from medium sized Tobacco".

This section specifies the definition, evaluation methods, and original tobacco quality requirements for high oil content tobacco leaves from medium sized Tobacco, which together with physical samples constitute the original tobacco quality requirements for high oil content tobacco leaves from Medium sized Tobacco.

This standard was proposed by the Standardization Management Committee of Hubei Tobacco Industry Co., Ltd. This standard was drafted and managed by the Technical Center.

The main drafters of this standard are He Jiewang, Dong Shiliang, Liu Fengfeng, Wang Jian, Shi Gang, Yan Tiejun, Si Hui, Liu Jianfeng, Wu Fengguang, Zhang Yaohua, Bao Jun, Wang Haiming, Wang Leopard Xiang, Wang Lin, Mou Yi, Zhang Jun, and Xie Lilei.

This standard was first published in December 2020.

High oil content tobacco leaves from medium sized tobacco - Part 1: Definition, evaluation methods, and quality requirements for raw tobacco

1 Range

This standard specifies the definition, evaluation methods, and original tobacco quality requirements for high oil content tobacco leaves from medium sized Tobacco. This standard is applicable to guide the production, raw tobacco procurement, and quality inspection of high oil tobacco leaves from medium sized Tobacco.

2 Normative reference documents

The contents of the following documents constitute essential clauses of this document through normative references in the text. Among them, for referenced documents with dates, only the version corresponding to that date is applicable to this document; The latest version (including all modifications) of the referenced document without a date is applicable to this document.

GB 2635 Tobacco

YC/T 31 Preparation of tobacco and tobacco products samples and determination of moisture Oven method

YC/T 159 Determination of water-soluble sugars in tobacco and tobacco products Continuous flow method

YC/T 160 Determination of total plant alkaloids in tobacco and tobacco products Continuous flow method

YC/T 161 Determination of total nitrogen in tobacco and tobacco products Continuous flow method

YC/T 162 Determination of chlorine in tobacco and tobacco products Continuous flow method

YC/T 216 Determination of Starch in Tobacco and Tobacco Products Continuous Flow Method

YC/T 217 Determination of Potassium in Tobacco and Tobacco Products Continuous Flow Method

3 Terminology and definitions

The following terms and definitions apply to this document.

3.1 Medium sized Smoke

It is a definition of the growth and appearance of tobacco plants in the field. Refers to adopting reasonable dense planting, balanced fertilization, and scientific methods in suitable ecological areas. Learn technical measures such as topping to promote the normal growth, development, and maturity of tobacco plants, coordinate the growth of tobacco plant populations and individuals, have a waist drum shaped or tube like plant shape, appropriate plant height, reasonable number of leaves, moderate length, width, and thickness of leaves, open top leaves, clear and uniform individual appearance, and obvious layering and yellowing.

3.2 High oil content

It is a definition of the richness of oil in roasted tobacco leaves. High oil tobacco leaves, with

a moisture content of 16% to 18%, appear oily, sticky, loose, soft, and uniformly bright. Specifically, it is characterized by a smooth and plump appearance, soft and sticky touch, elasticity, uniform color, bright luster, and good maturity.

3.3 Medium sized Tobacco High Oil Tobacco Leaves

It refers to tobacco leaves that have the characteristics of oil, softness, and brightness after baking, and have a moderate identity and size. It is a characteristic definition of the demand for high-quality tobacco leaves in the high-quality development of cigarettes, which meets the requirements of medium sized Tobacco. After baking, the tobacco leaves have high oil content, with a smooth and sticky appearance, loose and soft texture, uniform and bright color, moderate leaf identity and size, coordinated internal chemical composition, and sensory evaluation with aroma, sweetness, softness, and cleanliness.

3.4 Softness

Refers to the flexibility exhibited by tobacco leaves under external forces, reflecting the degree of softness, flexibility, and deformation of tobacco leaves. The softness index in appearance evaluation can refer to the test results of the softness meter (unit: mN). The lower the measured value of the softness meter, the better the softness of the leaf. Conversely, the larger the measured value of the softness meter, the worse the softness of the leaf.

3.5 Elastic

The degree and speed at which tobacco leaves recover to their original state after being stretched or compressed by external forces.

3.6 Gloss

The visual reflection of the difference between the bright and dark colors of tobacco leaves.

3.7 Color uniformity

The uniformity of color on the surface of tobacco leaves.

4 technical requirement

4.1 Formula positioning

Sensory evaluation has the characteristics of fragrance, sweetness, softness, and cleanliness, and is used for first class and above Huanghelou brand cigarette products to enrich cigarette aroma, improve smoke morphology, enhance satisfaction and comfort.

4.2 Appearance, leaf length, and single leaf weight

4.2.1 Appearance features

Moderate in identity and size, with characteristics of oil, softness, and brightness:

a) Oil: The oil appears to be moist and full, but feels sticky and full when touched, with a high to high oil content index;

b) Soft: Soft to the touch and sticky to the hand, with good maturity, a wrinkling sensation, and strong elasticity;
c) Bright: The color is bright yellow or golden, uniform in color, and the glossiness is bright; d) Moderate identity and size.

4.2.2 Indicator and score setting

The indicators and score settings are given in Table 1, with the minimum score unit being 1.

Table 1 Appearance evaluation indicators and score settings for original cigarettes

index	Score setting
Oil content (25)	23-25 more, 19-22 more, 13-18 more, 7-12 slightly, 1-6 less.
Identity (10)	Medium 8-10, slightly thinner 6-7, slightly thicker 3-5, thin or thick 1-2.
Maturity (15)	Mature 13-15, still mature 10-12, undercooked 7-9, false mature 1-6.
Softness (15)	Soft 13-15, relatively soft 10-12, less soft 7-9, hard and brittle 1-6.
Elasticity (5)	Strong 4-5, medium 3, weak 1-2.
Color (10)	Golden yellow, bright yellow 8-10, light yellow 6-7, dark yellow 4-6, red brown 1-3.
Color uniformity (10)	Uniform 8-10, relatively uniform 5-7, and less uniform 1-4.
Glossiness (10)	Bright 8-10, still bright 5-7, darker 1-4.

4.2.3 Indicator requirements

The requirements for the appearance, leaf length, and single leaf weight of high oil content tobacco leaves from Medium sized Tobacco are shown in Table 2.

Table 2: Requirements for Appearance, Leaf Length, and Single Leaf Weight of High Oil Tobacco Leaves

position		Quality factors									Leaf length (cm)	Single leaf weight (g)
		Oil content	identity	Maturity level	Softness	elastic	colour	Color is uniform Uniformity	gloss	total branch		
		twenty-five	ten	fifteen	fifteen	five	ten	ten	ten	one hundred		
upper part smoke	grade	More often	secondary	mature	Relatively soft	strong	Golden and straight yellow	uniformity	Bright and vibrant	/	45~70	9~17
	Minimum score	nineteen	eight	thirteen	eleven	five	ten	nine	eight	eighty-two		
in B u Y an	grade	More often	secondary	mature	soft	strong	Golden and straight yellow	uniformity	Bright and vibrant	/	55~70	8~15
	Minimum score	nineteen	eight	thirteen	thirteen	four	nine	eight	eight	eighty-eight		
Note: The specific indicators of suitable leaf length and single leaf weight for each production area are specified in the physical sample description.												

4.3 Sensory evaluation of suction quality

4.3.1 Sensory evaluation and absorption characteristics

Moderate concentration and vigor, with the characteristics of "fragrant, sweet, soft, and clean":

a) Fragrance: Full aroma, elegant texture, good translucency, sufficient fragrance, light impurities;

b) Sweet: There is a noticeable aftertaste in the mouth;
c) Soft: The smoke is soft, delicate, and smooth, with good clumping properties and slight irritation;
d) Clean: The aftertaste is clean and comfortable.

4.3.2 Indicator and score setting

The indicators and score settings are given in Table 3, with a minimum score unit of 0.5.

Table 3 Sensory evaluation indicators and score settings for smoking quality of single tobacco

index	Scoring criteria
Aroma quality (18)	Good 16-18, good 15-15.5, moderate+14-14.5, moderate 13-13.5, moderate -11-12.5, poor 9-15<br>10.5, with a difference of 0-8.5.
Aroma volume (16)	Foot 14.5-16, relatively foot 12.5-14, still full 1+10.5-12, still full 8.5-10, still sufficient -7.5-8<br>Less 5.5 to 7, less 0 to 5.
Miscellaneous Qi (16)	No 14.5-16, lighter 12.5-14, with+10.5-12, with 8.5-10, with -7.5-8, heavier 5.5-7<br>Weight 0-5.
Stimulating (20)	None 17.5-20, slightly 15.5-17,+13.5-15, 11.5-13, -9.5-11, larger 7.5-9<br>Big 0 to 7.
Aftertaste (22)	Comfortable 19.5-22, relatively comfortable 16.5-19+3~,+13.5-16, still comfortable 11.5-13, still comfortable -9.5~<br>11. Bitter and spicy 8.5~9, tongue stagnation 0~8.
Flammability (4)	Strong 3.5-4, moderate 2.5-3, poor 1.5+3.1, with 13-1
Grey (4)	White 3.5-4, gray white 2.5-3, gray 1.5+3.1 with 13-1
Concentration (5)	Concentrated 4.5-5, relatively concentrated 3.5-4, moderate+13-1 with. 5-16 comfortable 1.13, suitable-
Vigorous (5)	Big 4.5-5, Big 3.5-4, Moderate+13-1, Comfortable 5-16, Comfortable 1-13
Grouping (5)	Good 4.5 to 5, good 3.5 to 4, moderate+13 to 1, with 5 to 16 comfortable and 1 to 13 comfortable
Delicateness (5)	Delicate 4.5-5, relatively delicate 3.5-4, medium 15-15,+1 still 11.53, suitable for 9-0-1.
Sweet feeling (5)	Strong 4.5-5, Strong 3.5-4, Medium+13-1 with 13.56, Suitable for 1.53
Dry feeling (5)	Weak 4.5-5, Weak 3.5-4, Medium+13-1 with. 5-16 Comfort 1-13 Comfort

4.3.3 Indicator requirements

The sensory evaluation index requirements for high oil content tobacco leaves of Medium sizedTobacco are shown in Table 4.

Table 4 Requirements for sensory evaluation and smoking indicators of high oil content tobacco leaves in the original tobacco

	Quality characteris	Style features	Smoke characteristics	Taste characteristic

positi on		tics												s	
		aroma quali ty	aroma amo unt	Misc ellan eous Qi	stimul ate natur e	Aftertas te	burnin g natur e	ash colo ur	total bran ch	conce ntrati on	Vigor ousne ss	Form a grou p nature	exquisi te degree	Retur ning to sweet ness sens e	dry sense
		eight een	sixtee n	sixtee n	twent y	twenty-two	four	four	one hund red	five	five	five	five	five	five
uppe r part smoke	grade	prefer ably	Relati vely suffic ient	Light er	Micr o there	More comfort able	strong	whit e	/	Thick er	more	seco ndar y	seconda ry	secon dary	seco ndar y
	Minimum score	fiftee n	thirte en	thirte en	seven teen	sevente en	four	four	eight y-four	three point five	three point five	three	three	three	three
centr al secti on smoke	grade	good	Relati vely suffic ient	Light er	nothin g	More comfort able	strong	whit e	/	secon dary	mode rate	prefe rably	More delicate	Stron g	Wea ker
	Minimum score	sixtee n	fourte en	fourte en	eight een	eightee n	four	four	eight y-eight	three	three	four	four	four	four

4.4 Conventional chemical composition

The conventional chemical composition requirements for high oil content tobacco leaves from Zhongke Tobacco are shown in Table 5.

Table 5: Requirements for the Conventional Chemical Composition of High Oil Tobacco Leaves in Original Tobacco

position	nicotine	Total nitrogen	reducing sugar	total sugar	starch	Sugar alkali ratio
Upper smoke	2.5%～3.5%	1.8～2.8%	15%～26%	23%～33%	＜4.5%	7~15
Central Smoke	2.0%～3.0%	1.6～2.5%	20%～30%	25%～38%	＜4.5%	8~15
Note: The appropriate chemical composition content for each production area is specified in the physical standard sample description.						

4.5 water content

Moisture percentage: 16% to 18%.

5 sampling

5.1 The sampling quantity is 5-10kg, and an average of at least two samples are taken from all the samples opened during on-site inspection. If the number of opened samples exceeds 40, select 40 pieces.

Note: Same as clause 7.1.2 of GB 2635 "Tobacco".

5.2 Randomly select around 50 tobacco leaves from the sample for the detection of leaf length and single leaf weight.

6 Testing method

6.1 water content

Sensory inspection method for on-site inspection, oven inspection method for indoor inspection:

a) Sensory examination: The tobacco tendon is slightly soft and not easily broken, with a slight noise when held, and not easily broken;

b) Oven inspection: Conduct testing in accordance with the provisions of YC/T 31.

6.2 Appearance quality

6.2.1 Evaluation method

6.2.1.1 When evaluating, an evaluation team consisting of three or more quality inspectors should be established, with one team leader responsible for the orderly conduct of the entire evaluation process.

6.2.1.2 Evaluate according to the indicators and score settings given in 4.2.2, and record the data using the "Original Smoke Appearance Quality Evaluation Form" (See Appendix A), each indicator is scored in units of 1 point.

6.2.2 Result statistics

Calculate the average score of a single indicator according to equation (1), and keep the result to one decimal place.

$$\overline{X}_i = \frac{\Sigma X_i}{N}$$

In the formula: Xi -- average score of a single indicator;

Xi -- the sum of scores for a single indicator;

N -- Number of personnel participating in the evaluation.

6.3 Leaf length

After slightly flattening the tobacco leaves, use a ruler with a minimum scale of 0.1cm and a length greater than 100cm to measure. Pay attention to the force when flattening the tobacco leaves to avoid tearing them apart.

If the proportion of tobacco leaves that meet the requirements is not less than 85% of the total sampling, then the leaf length meets the requirements; otherwise, it does not meet the requirements.

6.4 Single leaf weight

Use a balance with an accuracy of not less than 0.1g for testing.

If the proportion of tobacco leaves that meet the requirements is not less than 85% of the total sampling, then the single leaf weight meets the requirements; otherwise, it does not meet the requirements.

6.5 Sensory evaluation of suction quality

6.5.1 Evaluation method

6.5.1.1

When evaluating, an evaluation team consisting of 5 or more evaluators should be established (with no less than 2 development and maintenance personnel for the leaf group formula), with one team leader responsible for the orderly conduct of the entire evaluation process.

6.5.1.2

Evaluate according to the indicators and score settings given in 4.3.2, and record the data using the "Single Material Smoke Sensory Evaluation Quality Evaluation Form" (see Appendix B), with each indicator scored in units of 0.5 points.

6.5.2 Result statistics

Execute according to clause 6.2.2 of this standard.

6.6 Conventional chemical composition

6.6.1 Nicotine

Conduct testing in accordance with YC/T 160 regulations.

6.6.2 Total nitrogen

Testing shall be conducted in accordance with the provisions of YC/T 161.

6.6.3 Total sugar and reducing sugar

Testing shall be conducted in accordance with the provisions of YC/T 159.

6.6.4 potassium

Testing shall be conducted in accordance with the provisions of YC/T 217.

6.6.5 chlorine

Conduct testing in accordance with YC/T 162 regulations.

6.6.6 starch

Conduct testing in accordance with YC/T 216 regulations.

6.7 Measurement method of softness meter

Randomly select 10 tobacco leaves, take the right side of the tobacco leaves, and take 2 cm × 2 cm small squares at the leaf tip (between the 3rd and 4th veins), middle (between the 6th and 7th veins), and base (between the 11th and 12th veins), avoiding the veins, for a total of 30 tobacco leaves. Use a constant temperature and humidity chamber to equilibrate for 48 hours under the conditions of temperature=(22 ± 1) ℃ and relative humidity=60% ± 2%. Use a softness tester to detect the average value of 30 tobacco leaves.
