# Supplementary material for: Metabolomics reveals signature metabolites associated with high-oiliness flue-cured tobacco (Nicotiana tabacum L.) leaves
Source: Front Plant Sci. 2026 Sep 4;17:1890505. doi: 10.3389/fpls.2026.1890505 (PMC13585713; doi:10.3389/fpls.2026.1890505)
Supplement: Supplementary file 1 [file DataSheet1.zip › Supplementary Figures.docx]

**Supplementary Figures**

**Figure S1**

**Figure S1.** QC sample evaluation chart

**Figure S2**

**Figure S2.** Sample Correlation heat map

**Figure S3**

**Figure S3.** Validation of the PLS-DA model by permutation testing (n = 200).

**Figure S4**

**Figure S4.** Receiver operating characteristic (ROC) curve analysis of candidate biomarker metabolites for discriminating oiliness levels in tobacco leaves. (A) Middle leaves; (B) Upper leaves.
